# Audit of high prevalent breast screening recall rates: Torbay Hospital

**DOI:** 10.1186/bcr3784

**Published:** 2015-11-05

**Authors:** Alexander Crowther, Rebecca Green

**Affiliations:** 1 Torbay DGH, Torquay, UK

## Introduction

The target percentage of women recalled after prevalent round breast screening is <7 % with minimum standards <10 %. Torbay Hospital's prevalent round recall is high at 11.4 %. We plan to assess patterns of recall by category to see if any particular reason for recall could be decreased.

## Methods

Retrospective audit of 12 months of prevalent round recalls March 2013-February 2014. All age groups were included. Each recall was grouped into one/more of the following categories: calcification, well-defined mass, ill-defined mass, asymmetric density, distortion, clinical, other. We will calculate the proportion of recalls per group that proved to be malignancy and assess to see if any category was a poor predictor of malignancy. All histology proven malignancies from 2012/13 and 2014/15 will also be categorised by group.

## Results

There were 215 recalls for ages 49–69, 15 proven malignancies. 77% of Ill-defined mass, 22% of distortion and 10% of calcifications recalled proved to be malignant and are the strongest predictors of malignancy. Well-defined mass and asymmetric density had 0 % malignancy rates and accounted for 129 (59.4 %) of prevalent recalls. Thirteen clinical recalls (1.4 %) were also 0 % for malignancy but beyond the control of the screening service. Further audit was performed looking at the proven malignancies from 2012/13 and 2014/15, which showed a total of 33 malignancies with 13 calcifications, 17 ill-defined masses, one asymmetry, one distortion and one clinical recall.

## Conclusion

A high proportion of recalls (60 %) are for well-defined mass and asymmetric density which have poor predictive outcome. These groups are potential areas to decrease recall rates. A total 1.4 % of clinical recalls are beyond the control of the screening service, which would bring prevalent recalls to a compliant level of 10 %.

